# Helmet use among Alaskan children involved in off-road motorized vehicle crashes

**DOI:** 10.3402/ijch.v73.25191

**Published:** 2014-09-16

**Authors:** Christopher W. Snyder, Oliver J. Muensterer, Frank Sacco, Shawn D. Safford

**Affiliations:** 1 Department of Surgery, 6th Medical Group, MacDill Air Force Base, Tampa, FL, USA; 2 Department of Surgery, The Children's Hospital at Providence Alaska Medical Center, Anchorage, AK, USA; 3 Division of Acute Care Surgery, University of South Florida, Tampa, FL, USA; 4 Division of Pediatric Surgery, The Children's Hospital at Montefiore, Albert Einstein College of Medicine, Bronx, NY, USA; 5 Department of Surgery, Alaska Native Medical Center, Anchorage, AK, USA; 6 Division of Pediatric Surgery, Virginia Tech Carilion School of Medicine, Roanoke, VA, USA

**Keywords:** helmet, traumatic brain injury, all-terrain vehicle, snowmobile, motorbike, Alaska

## Abstract

**Background:**

Off-road motorized vehicle crashes are a common source of trauma among Alaska children. Injury morbidity is worse in Alaska Native children than non-Native children, but the reasons are unclear.

**Objective:**

To evaluate the differences in helmet use between the Native and the non-Native children, and to assess the impact of helmet use on injury patterns and outcomes.

**Design:**

This retrospective cohort study identified patients aged 17 or younger admitted after all-terrain vehicle, snowmobile or motorbike injury between 2001 and 2011 from the Alaska Trauma Registry. Helmeted and non-helmeted patients were compared with respect to demographics, central nervous system (CNS) injury and the overall risk of death or permanent disability. Logistic regression was used to evaluate predictors of helmet use and the effects of ethnicity and helmet use on outcomes.

**Results:**

Of the 921 injured children, 51% were Alaska Native and 49% were non-Native. Helmet use was lower among Native versus non-Native patients on unadjusted comparison (24% vs. 71%) and multivariable logistic regression (OR 0.17, 95% CI 0.11–0.27, p<0.0001). Prevalence of CNS injury was higher among Native children (39.7% vs. 30.4%, p=0.016). However, on logistic regression with adjustment for helmet use, Native ethnicity was not a significant predictor of CNS injury (OR 1.07, 95% CI 0.68–1.68, p=0.78), whereas helmet use was strongly protective against CNS injury (OR 0.28, 95% CI 0.18–0.44, p<0.0001) as well as death or permanent disability (OR 0.26, 95% CI 0.10–0.67, p=0.006).

**Conclusions:**

Helmet use is lower among Alaska Native children involved in off-road motorized vehicle crashes. These ethnic disparities in helmet use contribute to higher rates of CNS injury among Native children. Helmet use significantly improves overall outcome. Helmet promotion efforts should be expanded, especially in Native communities.

Injuries from off-road motorized vehicles are a significant source of morbidity and mortality in children and adolescents. Such vehicles include snowmobiles, motorbikes and all-terrain vehicles (ATVs). Wearing an appropriate protective helmet has been strongly advocated as a means of secondary injury prevention. The rising incidence and severity of injuries from these vehicles has prompted multiple professional organizations to issue recommendations restricting their use in the paediatric population (1–5).

In Alaska, the largest and the northernmost state in the United States, a large proportion of the population lives in rural areas with limited road access. Many people, including children, use off-road motorized vehicles as a primary mode of transportation. Alaska's off-road vehicle-related mortality is significantly higher than other states and has disproportionately high impact on people of Alaska Native ethnicity (6,7). Low rates of helmet use have been reported in certain Alaskan Native communities (8), but helmet use in the wider Alaskan paediatric population has not been well-defined. The purpose of this study was to investigate factors associated with helmet use among Alaskan children involved in off-road motorized vehicle crashes and to determine the effects of helmet use on injury patterns and outcomes.

## Methods

Records of traumatic injuries sustained between January 2001 and December 2011 by children aged 0–17 were obtained from the State of Alaska Trauma Registry. The registry has been described in detail elsewhere (9). Off-road motorized vehicle injuries were identified by Centers for Disease Control and Prevention (CDC) E-codes. Off-road motorized vehicles were defined as ATVs (E821.0, 821.1, 821.8), snowmobiles (E820.0, 820.1, 820.8, 820.9) and motorbikes (E812–819[.2], 821–825[.2]). The brief narrative injury description for each record was checked to verify accuracy of the E-code assignment. Trivial injuries with Injury Severity Score (ISS) less than or equal to one were excluded.

Helmet use, demographics, recent alcohol or drug use (within 6 hours of injury) and injury characteristics were obtained from the registry. The registry captures up to 10 International Classification of Diseases, Ninth Revision (ICD-9) codes for specific injuries. Injury patterns were derived from these ICD-9 codes as follows: central nervous system (CNS) injury (800–804, 850–854, 870), thoracic (860–862), abdominal solid organ (864–866, 868.01, 868.11, 863.81–84 and .91–94), abdominal other (863, 867–869 [except as previously specified]), pelvic fracture (808) and vascular (900–904). The zip code of injury location was linked with publicly available Rural-Urban Commuting Area (RUCA) codes to classify the community type in which the injury occurred. RUCA codes are a classification scheme based on census tract that characterizes communities by combining standard Bureau of Census Urbanized Area and Urban Cluster definitions with work commuting information (10). Based on the RUCA code, communities were classified as urban (1.0, 1.1, 2.0, 2.1, 3.0, 4.1, 5.1, 7.1, 8.1, 10.1), large rural town (4.0, 4.2, 5.0, 5.2, 6.0, 6.1) or small, isolated rural town (7.0, 7.2, 7.3, 7.4, 8.0, 8.2, 8.3, 8.4, 9.0, 9.1, 9.2, 10.0, 10.2, 10.3, 10.4, 10.5, 10.6). The primary outcomes of interest were CNS injury and overall unfavourable outcome (either death or permanent disability). Secondary outcomes included ISS and risk of thoracoabdominal, pelvic or vascular injury.

Patients were initially divided into 3 groups based on helmet use at the time of injury: helmeted, not helmeted or helmet status unknown. Demographics, injury characteristics and outcomes for these groups were compared with chi-square and Kruskal–Wallis tests for categorical and continuous variables, respectively. Logistic regression modelling was used to identify variables predictive of helmet use. Prevalence of CNS injury was compared between Native and non-Native children using the chi-square test. Another logistic regression model was developed to evaluate the effects of helmet use on the likelihood of CNS injury, with adjustment for relevant covariates and assessment for interactions between variables. Models were developed for unfavourable outcome and thoracoabdominal/pelvic/vascular injury in similar fashion. Generalized linear modelling was used to evaluate the effect of helmet use on ISS. Regression models were developed using complete cases only; records with unknown helmet status were excluded.

To address the possibility of biased parameter estimates due to the relatively large proportion of missing data, multiple imputation was performed in accordance with Rubin and Schenker (11). Ten imputations were performed. The previously constructed regression models were repeated, using the imputed datasets. The parameter estimates obtained from the imputed analysis were then compared with those obtained from the complete case analysis. All statistical analysis was performed with SAS 9.2 (SAS Institute, Cary, NC).

## Results

Of the 921 patients meeting the inclusion criteria, 301 (33%) were helmeted, 352 (38%) were non-helmeted and in 268 (29%) the helmet status was missing. Descriptive statistics for each group are provided in Table I. Ethnicity was Alaska Native/American Indian (Native) for 467 patients (51%) and White/other (non-Native) for 454 patients (49%).

**Table I:** Descriptive statistics for paediatric off-road motorized vehicle crashes, by helmet status*

	Helmet (N=301)	No helmet (N=352)	Helmet status unknown (N=268)
Age, years	15 (13–16)	14 (11–16)	14 (11–16)
Female sex	68 (23)	134 (38)	69 (26)
Ethnicity			
Alaska Native/American Indian	81 (27)	262 (74)	124 (46)
White	197 (65)	79 (22)	130 (49)
Other/unknown	23 (8)	11 (3)	14 (5)
Community type			
Urban	143 (48)	69 (20)	97 (36)
Large rural	31 (10)	12 (3)	11 (4)
Small, isolated rural	127 (42)	271 (77)	160 (60)
Vehicle type			
All-terrain vehicle (ATV)	112 (37)	234 (66)	138 (52)
Snowmachine	86 (29)	92 (26)	76 (28)
Motorbike	103 (24)	26 (7)	54 (20)
Injury Severity Score (ISS)	9 (4–9)	6 (4–10)	4 (4–9)
ISS≥10	72 (24)	94 (27)	31 (12)
Central nervous system (CNS) injury	43 (14)	117 (33)	32 (12)
Any thoracoabdominal/pelvic/vascular injury	68 (23)	29 (8)	41 (15)
Thoracic	34 (11)	19 (5)	12 (4)
Abdominal – solid organ	36 (12)	9 (3)	28 (10)
Abdominal – other	3 (1)	2 (1)	0
Pelvic fracture	14 (5)	13 (4)	7 (3)
Vascular injury	5 (2)	2 (1)	2 (1)
Combined CNS & thoracoabdominal/pelvic/vascular injury	6 (2)	11 (3)	2 (1)
Death	4 (1)	6 (2)	3 (1)
Death or permanent disability	12 (4)	21 (6)	5 (2)

* Categorical variables presented as N (%); continuous variables as median (interquartile range).

Among children with known helmet status, rates of helmet use were 24 and 71% for Native and non-Native children, respectively. Results of the final logistic regression model for likelihood of helmet use are shown in Table II. Variables independently associated with lower likelihood of helmet use were Alaska Native/American Indian ethnicity, younger age, small rural community location and recent alcohol use. Helmet use was also significantly less likely among ATV users compared to snowmobile and motorcycle users.

**Table II:** Logistic regression model for likelihood of helmet use at time of off-road motorized vehicle crash

Variable	Odds ratio	95% Confidence interval	p
Vehicle type			
ATV	1.0	–	–
Snowmachine	2.94	1.85–4.68	<0.0001
Motorbike	6.60	3.72–11.72	<0.0001
Age (per year increase)	1.08	1.01–1.15	0.025
Male sex	1.35	0.87–2.09	0.184
Ethnicity			
White/other	1.0	–	–
Alaska Native/American Indian	0.17	0.11–0.27	<0.0001
Community type			
Urban	1.0	–	–
Large rural	2.08	0.90–4.80	0.086
Small, isolated rural	0.55	0.35–0.88	0.012
Recent alcohol use	0.25	0.11–0.59	0.002
Recent drug use	1.17	0.45–2.99	0.749

**Fig. 1:**
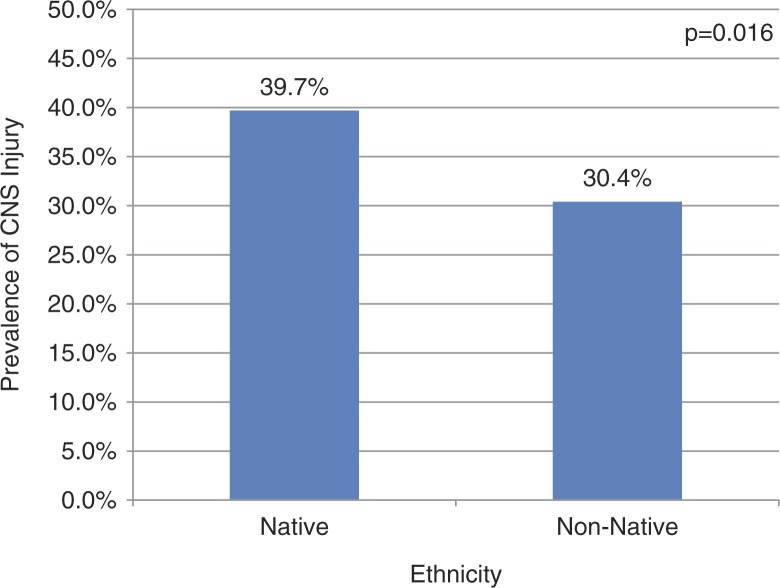
Prevalence of CNS injury after off-road motorized vehicle crash among Alaskan children by ethnicity.

On unadjusted comparison, the prevalence of CNS injury was significantly higher among Native versus non-Native children (39.7% vs. 30.4%, p=0.016) (Fig. 1). However, on multiple regression modelling, Native ethnicity no longer had any effect (Table III). Helmet use was strongly protective against CNS injury (OR 0.28, 95% CI 0.18–0.44, p<0.0001). Conversely, recent drug use was associated with a nearly 7-fold higher risk of injury to the CNS.

**Table III:** Logistic regression model for likelihood of CNS injury after off-road motorized vehicle crash

Variable	Odds ratio	95% Confidence interval	p
Helmet use	0.28	0.18–0.44	<0.001
Vehicle type			
ATV	1.0	–	–
Snowmachine	0.60	0.39–0.93	0.02
Motorbike	0.75	0.44–1.29	0.30
Age (per year increase)	0.98	0.93–1.03	0.17
Male sex	0.96	0.65–1.41	0.84
Ethnicity			
Alaska Native/American Indian	1.0	–	–
White/other	1.07	0.68–1.68	0.78
Community type			
Urban	1.0	–	–
Large rural	1.10	0.52–2.34	0.81
Small, isolated rural	0.75	0.47–1.19	0.22
Recent alcohol use	0.94	0.48–1.83	0.85
Recent drug use	6.58	2.92–14.92	<0.001

The effect of helmet use on the likelihood of death or permanent disability is shown in Table IV. Helmet use was associated with a significantly lower likelihood of death or permanent disability (OR 0.26, 95% CI 0.10–0.67, p=0.006). Interactions between helmet use and other variables were assessed and found to be non-significant, indicating that the protective effect of helmet use was relatively uniform across vehicle types. Risk of unfavourable outcome was significantly higher after motorcycle injuries compared to ATV injuries (OR 3.41, 95% CI 1.28–9.10, p=0.015).

**Table IV:** Logistic regression model for likelihood of death or permanent disability after off-road motorized vehicle crash

Variable	Odds ratio	95% Confidence interval	p
Helmet use	0.26	0.10–0.67	0.006
Vehicle type			
ATV	1.0	–	–
Snowmachine	0.49	0.14–1.76	0.27
Motorbike	3.41	1.28–9.10	0.02
Age (per year increase)	1.05	0.92–1.20	0.46
Male sex	0.47	0.21–1.07	0.07
Ethnicity			
White/other	1.0	–	–
Alaska Native/American Indian	0.44	0.17–1.11	0.08
Community type			
Urban	1.0	–	–
Large rural	1.70	0.42–6.90	0.46
Small, isolated rural	0.97	0.38–2.46	0.94
Recent alcohol use	0.71	0.17–2.92	0.64
Recent drug use	2.00	0.55–7.25	0.29

Regression modelling for secondary outcomes revealed that helmet use was associated with a higher risk of thoracoabdominal, pelvic or vascular injury (OR 2.33, 95% CI 1.35–4.05, p=0.003). This higher risk was driven by a higher incidence of abdominal solid organ injuries among helmeted riders (12% vs. 3%). Helmet use had no significant effect on overall ISS.

When multiple imputation was performed and compared with complete case analysis, there were minimal differences in the magnitude of the parameter estimates, but no differences in the direction or statistical significance of variable effects. After performing multiple imputation, helmet use remained similarly protective against death or permanent disability (OR 0.31, 95% CI 0.13–0.76, p=0.010).

## Discussion

This study found that helmet use is strongly associated with better overall outcomes in Alaskan children sustaining off-road motorized vehicle injuries. These superior outcomes appear to be driven by a reduction in the risk of CNS injury. These findings are consistent with recent literature showing higher morbidity associated with CNS injury, and a lower risk of CNS injury with helmet use (12–16). The higher risk of abdominal solid organ injury among helmeted riders may explain why this study and other studies did not show a reduction in overall ISS with helmet use (17). The mechanism whereby helmet use would increase abdominal solid organ injury is unclear. It is possible that helmet use alters crash mechanics in such a way as to make the abdomen more vulnerable to injury. Some have postulated a similar mechanism for cervical spine injury with helmet use, although such arguments have been largely discredited (18).

This study also defined specific risk factors for helmet non-use. Children of Alaska Native ethnicity are over 5 times less likely than children of other ethnicities to be wearing a helmet. This stark disparity in helmet use may explain some of the disparities seen when comparing trauma outcomes between Native and non-Native populations (19,20). Although our data showed a significant difference in prevalence of CNS injury between Native and non-Native children, this difference disappeared when helmet status was taken into account.

We also found that children from more remote communities are also less likely to wear helmets, regardless of ethnicity. Similarly, Su et al. found an 84% rate of helmet non-use among Canadian children injured in ATV accidents, with even higher rates of non-use in remote communities (21). Previous work has demonstrated that user-reported barriers to helmet use include user discomfort, inconvenience and lack of perceived risk for non-use (22). Such barriers may be applicable in the Alaskan population as well. Since off-road vehicles often serve as the primary mode of transportation in remote communities with limited road systems, they are not considered “recreational” vehicles. Their familiarity and use for everyday tasks may make such vehicles seem less hazardous to the community at large (23). The barrier of inconvenience may also explain why younger children were less likely than older children to be helmeted. Acquiring appropriately child-sized helmets may be difficult for a family living in a remote village. The association of recent alcohol use with helmet non-use is expected, given the well-known effects of substance use on risky behaviours. This finding underscores the need for ongoing efforts to prevent substance abuse in both children and adults.

Our findings must be interpreted in light of some limitations. As with most large retrospective studies, our results may be biased due to misclassified or missing data. We sought to minimize such bias by manually verifying each injury E-code against the narrative injury description and by performing confirmatory multiple imputation. Uncontrolled confounding factors may also be present, since helmet use may be a marker for safer riding behaviours in general rather than a sole cause of benefit. It is also possible that systematic biases may result in a higher incidence of missed CNS injury among Native patients. However, if present, such biases would be towards the null and would actually strengthen the conclusions of the study.

Since the study is registry-based and not population-based, its findings must be applied with caution to the wider Alaskan paediatric population. For example, the study cannot be used to estimate overall rates of helmet use in the Alaskan paediatric population. The prevalence of helmet use in this study is likely lower than that of the general paediatric population, since only patients who were significantly injured (and thus presumably less likely to be helmeted) were included. This study also likely underestimates the magnitude of protective effect of helmet use. Since many helmeted patients who crash may not be injured at all, they will never present for medical care and never be entered into the registry. Conversely, some unhelmeted patients may sustain such severe injuries that they die in the field and are also never entered into the registry.

Preventing paediatric off-road vehicle injury requires a multifaceted approach. Restrictive laws and regulations, while an important component of prevention must be culturally appropriate and consistent with the needs of the community in order to be enforceable and effective (24–26). Efforts to modify off-road vehicle designs using safety engineering principles are currently underway, with a goal of making them inherently safer and more stable in the future (27). Design modifications to enhance the comfort and effectiveness of children's helmets may also be useful. Community education, targeted to an appropriate audience, is also an essential facet of paediatric injury prevention. Our study is especially useful for enhancing such educational efforts in Alaska. It demonstrates a clear outcome benefit with helmet use in an Alaskan paediatric population, lending further credibility to existing helmet education programmes. More importantly, it allows such helmet promotion and education efforts to be further targeted for groups that are particularly prone to helmet non-use. Future studies should investigate the effectiveness of helmet promotion and other strategies in helping enhance the health and safety of Alaska's children.

## References

[1] Burd R (2009). American Pediatric Surgical Association Trauma Committee position statement on the use of all-terrain vehicles by children and youth. J Pediatr Surg.

[2] American Academy of Pediatrics (2000). All-terrain vehicle injury prevention: two-, three-, and four-wheeled unlicensed motor vehicles. Pediatrics.

[3] American Academy of Pediatrics Committee on Injury and Poison Prevention (2000). Snowmobiling hazards. Pediatrics.

[4] Trauma Committee of the Canadian Association of Pediatric Surgeons (2008). Canadian Association of Pediatric Surgeons’ position statement on the use of all-terrain vehicles by children and youth. J Pediatr Surg.

[5] Yanchar NL (2012). Preventing injuries from all-terrain vehicles. Paediatr Child Health.

[6] Helmkamp JC, Aitken ME, Graham J, Campbell CR (2012). State-specific ATV-related fatality rates: an update in the new millennium. Public Health Rep.

[7] Hill R, Wells RS, Andon H, Ballew C (2004). Non-fatal injury hospitalizations among Alaska natives, 1994–1999: results from the Alaska Trauma Registry. Alaska Med.

[8] Redwood DG, Hagan KD, Perkins RD, Stafford HB, Orell LJ, Lanier AP (2009). Safety behaviours among Alaskan Native and American Indian people living in Alaska. Inj Prev.

[9] Kilkenny SJ, Moore MA, Simonsen BL, Johnson MS (1992). The Alaska trauma registry. Alaska Med.

[10] Rural-Urban Commuting Area Codes (RUCAs). http://depts.washington.edu/uwruca/.

[11] Rubin DB, Schenker N (1991). Multiple imputation in health-care databases: an overview and some applications. Stat Med.

[12] Ganti L, Bodhit AN, Daneshvar Y, Patel PS, Pulvino C, Hatchitt K (2013). Impact of helmet use in traumatic brain injuries associated with recreational vehicles. Adv Prev Med.

[13] Rostas JW, Donnellan KA, Gonzalez RP, Brevard SB, Ahmed N, Rogers EA (2014). Helmet use is associated with a decrease in intracranial hemorrhage following all-terrain vehicle crashes. J Trauma Acute Care Surg.

[14] Sandler G, Soundappan SS, Manglick MP, Fahy FE, Ross F, Lam L (2012). Pediatric “off-road vehicle” trauma: determinants of injury severity and type. Pediatr Emerg Care.

[15] Pelletier JS, McKee J, Ozegovic D, Widder S (2012). Retrospective review of all-terrain vehicle accidents in Alberta. Can J Surg.

[16] Ramakrishnaiah RH, Shah C, Parnell-Beasley D, Greenberg BS (2013). Motorized dirt bike injuries in children. J Emerg Med.

[17] Gittelman MA, Pomerantz WJ, Groner JI, Smith GA (2006). Pediatric all-terrain vehicle-related injuries in Ohio from 1995 to 2001: using the injury severity score to determine whether helmets are a solution. Pediatrics.

[18] Crompton JG, Bone C, Oyetunji T, Pollack KM, Bolorunduro O, Villegas C (2012). Motorcycle helmets associated with lower risk of cervical spine injury: debunking the myth. J Am Coll Surg.

[19] Bazarian JJ, Pope C, McClung J, Cheng YT, Flesher W (2003). Ethnic and racial disparities in emergency department care for mild traumatic brain injury. Acad Emerg Med.

[20] American Academy of Pediatrics (1999). Committee on Native American Child Health and Committee on Injury and Poison Prevention. The prevention of unintentional injury among American Indian and Alaska Native children: a subject review. Pediatrics.

[21] Su W, Hui T, Shaw K (2006). All-terrain vehicle injury patterns: are current regulations effective?. J Pediatr Surg.

[22] Adams LE, Aitken ME, Mullins SH, Miller BK, Graham J (2013). Barriers and facilitators to all-terrain vehicle helmet use. J Trauma Acute Care Surg.

[23] Miller M, Davidov D, Tillotson R, Whiteman C, Marshall T, Lander O (2012). Injury prevention and recreational all-terrain vehicle use: the impact of helmet use in West Virginia. W V Med J.

[24] McBride AS, Cline DM, Neiberg RH, Westmoreland KD (2011). Pediatric all-terrain vehicle injuries: does legislation make a dent?. Pediatr Emerg Care.

[25] Berger LR, Wallace LJ, Bill NM (2009). Injuries and injury prevention among indigenous children and young people. Pediatr Clin North Am.

[26] Winfield RD, Mozingo DW, Armstrong JH, Hollenbeck JI, Richards WT, Martin LC (2010). All-terrain vehicle safety in Florida: is legislation really the answer?. Am Surg.

[27] Jennissen CA, Miller NS, Tang K, Denning GM (2014). Optimising seat design for all-terrain vehicle injury prevention: wide variability illustrates need for evidence-based standardisation. Inj Prev.

